# Systemic translocation of *Staphylococcus* drives autoantibody production in HIV disease

**DOI:** 10.1186/s40168-019-0646-1

**Published:** 2019-02-14

**Authors:** Zhenwu Luo, Min Li, Yongxia Wu, Zhefeng Meng, Lisa Martin, Lumin Zhang, Elizabeth Ogunrinde, Zejun Zhou, Shenghui Qin, Zhuang Wan, Maria Anna Julia Westerink, Stephanie Warth, Hui Liu, Ping Jin, David Stroncek, Quan-Zhen Li, Ena Wang, Xueling Wu, Sonya L. Heath, Zihai Li, Alexander V. Alekseyenko, Wei Jiang

**Affiliations:** 1 Department of Microbiology and Immunology, Medical University of South Carolina, 173 Ashley Ave. BSB208D, Charleston, SC 29425 USA; 2 Department of Gastroenterology, Oncology Bioinformatics Center, Minhang Hospital, Fudan University, Shanghai, China; 3 Division of Infectious Diseases, Department of Medicine, Medical University of South Carolina, Charleston, SC 29425 USA; 4 Department of Medicine, Icahn School of Medicine at Mount Sinai, New York, NY 10029 USA; 5 Cell Processing Section (CPS), Department of Transfusion Medicine, Clinical Center, NIH, Bethesda, 20892 USA; 6 Department of Immunology and Internal Medicine, University of Texas Southwestern Medical Center, 5323 Harry Hines Blvd, Dallas, TX 75390 USA; 7 Sidra Medical and Research Center, Doha, Qatar; 8 Aaron Diamond AIDS Research Center, The Rockefeller University, New York, NY 10016 USA; 9 Division of Infectious Diseases, Department of Medicine, University of Alabama at Birmingham, Birmingham, AL 35294 USA; 10 Program for Human Microbiome Research, Biomedical Informatics Center, Department of Public Health Sciences, Medical University of South Carolina, Charleston, SC 29425 USA

**Keywords:** Autoantibodies, *Staphylococcus*, Plasma microbial 16S rDNA

## Abstract

**Background:**

Increased autoreactive antibodies have been reported in HIV disease; however, the mechanism accounting for autoantibody induction in HIV remains unknown.

**Results:**

Herein, we show that seasonal influenza vaccination induces autoantibody production (e.g., IgG anti-nuclear antibody (ANA) and anti-double-stranded DNA antibody (anti-dsDNA)) in some viral-suppressed antiretroviral therapy (ART)-treated HIV+ subjects, but not in healthy controls. These autoantibodies were not derived from antigen-specific B cells but from activated “bystander” B cells analyzed by single-cell assay and by study of purified polyclonal ANAs from plasma. To explore the mechanism of autoantibody generation in HIV+ subjects, plasma level of microbial products, gene expression profile of B cells, and B cell receptor (BCR) repertoires were analyzed. We found that autoantibody production was associated with increased plasma level of microbial translocation; the patients with high autoantibodies had skewed B cell repertoires and upregulation of genes related to innate immune activation in response to microbial translocation. By analyzing circulating microbial 16S rDNA in plasma, the relative abundance of *Staphylococcus* was found to be associated with autoantibody production in HIV+ subjects. Finally, we found that injection of heat-killed *Staphylococcus aureus* promoted germinal center B cell responses and autoantibody production in mice, consistent with the notion that autoantibody production in HIV+ patients is triggered by microbial products.

**Conclusions:**

Our results showed that translocation of *Staphylococcus* can promote B cell activation through enhancing germinal center response and induces autoantibody production. It uncovers a potential mechanism linking microbial translocation and autoimmunity in HIV+ disease and provides a strong rationale for targeting *Staphylococcus* to prevent autoantibody production.

**Electronic supplementary material:**

The online version of this article (10.1186/s40168-019-0646-1) contains supplementary material, which is available to authorized users.

## Introduction

In healthy people, most autoreactive B cells are effectively removed from the germinal center (GC), and only a small portion of self-reactive GC B cells can escape from deletion [1]. In HIV+ individuals, many autoantibodies are reported and autoimmune diseases occur during immunological recovery after antiretroviral therapy (ART) [2–4]. ART treatment significantly controls viral replication, decreases chronic immune activation, and partially recovers intestinal mucosal integrity in HIV disease [5]. ART-treated patients suffer from autoimmune symptoms associated with increased levels of B cell activation, pathologic autoantibodies, and residual systemic microbial translocation [6, 7].

Although many pathophysiologic hypotheses have been posited on the synthesis of autoantibodies, such as a direct role of viral particles, molecular mimicry, immune complex, polyclonal B lymphocyte activation, and dysregulation of B/T lymphocyte interaction [8–11], how autoantibody is produced during immunological recovery under ART remains unknown. In ART-treated HIV+ subjects, the immune responses (e.g., CD4+ T cell function) mostly recover, but the humoral immune system is subject to repeated and long-term stimulation through systemic bacterial products (e.g., LPS) [12]. Microbial translocation has been linked to HIV-associated B cell hyperactivation and perturbation [13]. Toll-like receptor (TLR) ligands play a role in B cell perturbation, autoantibody production, and autoimmune diseases [14–18]. In the current study, we found autoantibodies were induced in response to influenza vaccination in some viral-suppressed ART-treated HIV+ individuals. Moreover, vaccine-mediated autoantibody production was associated with baseline increased plasma level of microbial translocation and relative enrichment of translocation of *Staphylococcus* in HIV+ subjects but not in healthy controls. Furthermore, GC B cell activation and autoantibody induction were observed in C57BL/6 mice after intraperitoneal injection of heat-killed *Staphylococcus aureus*.

## Materials and methods

### Study subjects

In the current study, 16 healthy controls and 26 aviremic ART-treated HIV+ subjects were included. HIV-infected subjects were on viral-suppressive ART for at least 2 years. Inclusion criteria were as follows: (1) men and women age 18 or older, (2) be able and willing to provide informed consent, (3) healthy control individuals have self-reports of HIV-negative, and (4) HIV+ individuals have been treated with ART and have plasma HIV RNA below the limit of detection for at least 24 weeks (a single blip to ≤ 500 copies/mL will be allowed). Exclusion criteria were as follows: (1) pregnancy or breast-feeding by self-report; (2) recent severe illness such as anemia; (3) need for or use of specific medications during the 120 days prior to enrollment: antibiotics, systemic immunomodulatory agents, and supraphysiologic doses of steroids (> 10 mg/day); and (4) any other condition that the investigator judge makes the subject unsuitable for the study or unable to comply with the study requirements. The clinical characteristics of patients are shown in Additional file 1: Table S1. Participants received a single intramuscular dose of inactivated trivalent vaccine (2013-2014, Fluvirin, GSK, Philadelphia, PA), containing 15 μg influenza hemagglutinin (HA) of each strain of A/Christchurch/16/2010 (H1N1), A/Texas/50/2012 (H3N2), and B/Massachusetts/2/2012. This study was approved by the Medical University of South Carolina (Pro00020606).

### Mice

C57BL/6 mice were purchased from the Jackson Laboratories and housed at the Medical University of South Carolina. All animal studies were approved by the Institutional Animal Care and Use Committee (IACUC) at the Medical University of South Carolina. Mice were injected with PBS, heat-killed *Salmonella typhimurium* (HKST, InvivoGen, San Diego, CA), heat-killed *Pseudomonas aeruginosa* (HKPA, InvivoGen), or heat-killed *Staphylococcus aureus* (HKSA, InvivoGen) twice a week for 4 weeks and then once a week for 8 weeks by intraperitoneal (i.p.) route. The heat-killed bacteria were given 5 × 10^7^/mice/time.

### Flow cytometric analysis of cells from mice

Mononuclear cells were obtained from mouse spleen or lymph nodes by physical digestion and straining through a 70-μm filter, and stained for surface markers and intracellular cytokines using standard flow cytometric protocols. The following antibodies were used for cell staining: anti-CD3-PerCP-Cy5.5 (17A2), anti-CD4-BV510 (RM4-5), anti-CD8a-APC-vio770 (53-6.7), anti-CD44-FITC (IM7), anti-CD62L-BV421 (MEL-14), anti-CD25-PE-vio770 (7D4), anti-CD69-PE (H1.2F3), anti-IL-17A-PE (TC11-18H10), anti-IL-22-APC (IL22JOP), anti-IFN-γ-PE-Cy7 (XMG1.2), anti-CD19-BV421 (1D3), anti-B220-PerCP-cy5.5 (RA3-6B2), anti-GL7-PE (GL7), anti-CD95-PE-vio770 (REA453), anti-CD86-APC-vio770 (PO3.3), goat anti-mouse IgG-FITC, and anti-IgM-BV510 (R6-60.2). For anti-IFN-γ staining, cells were stimulated in complete RPMI-1640 + 10% FBS with leukocyte activation cocktail (BD, San Jose, CA) at 2 μL/mL. After being cultured at 37 °C for 4 h, cells were collected and washed with PBS. Fifty microliters of aqua blue (Life Technologies, Carlsbad, CA) was used at 4 °C for 20 min to exclude dead cells, then surface markers and intracellular cytokines were used by standard flow cytometric protocols. Cells were collected in a BD FACSVerse flow cytometer (BD, San Jose, CA), and data were analyzed by FlowJo software (version 10.0.8).

### Flow cytometric analysis of cells from human

Plasma was separated from EDTA-contained fresh blood samples, aliquoted, and stored at − 80 °C. Peripheral blood mononuclear cells (PBMCs) were isolated over a Ficoll-Paque cushion (GE Healthcare, Wauwatosa, WI). PBMCs were used for annexin V assays. Blood samples were used for all other flow cytometry-based assays except annexin V assays. For surface staining, antibodies were incubated with blood or PBMCs at room temperature for 15 min. After surface staining in blood samples, red cells were lysed, washed, and analyzed by flow cytometry. The fluorochrome-labeled mAbs (BD Pharmingen, San Jose, CA) used for flow cytometry included the following: anti-human CD3 (OKT3), anti-human CD4 (RPA-T4), anti-human CD8 (RPA-T8), anti-human CD19 (HIB19), anti-human CD20 (L27), anti-human CD27 (M-T271), anti-human CD38 (HIT2), anti-human CD45RA (HI100), anti-human HLA-DR (G46-6), anti-human ki67 (B56), anti-human IgD (IA6-2), anti-human IgG (G18-145), isotype control antibodies (BD Pharmingen), and annexin V (BD Pharmingen). Cells were collected in a BD FACSVerse flow cytometer (BD, San Jose, CA), and data was analyzed by FlowJo software (Version 10.0.8).

### ANA and anti-dsDNA antibody detection

Plasma levels of anti-dsDNA IgG and IgM were quantified using a commercial kit according to the manufacturer’s protocol (Immuno-Biological Laboratories, Minneapolis, MN). Antinuclear antibody (ANA) IgG detection was performed in plasma by ELISA using Hep-2 laryngeal carcinoma cell lysates as the coating antigens (ATCC, Manassas, VA).

### Sorting of antigen-specific single B cells by flow cytometry

The method of antigen-specific single B cell sorting was described in a previous study [19]. In the current study, 80–200 bp dsDNAs from calf thymus were purified by gel electrophoresis and biotinylated according to the manufacturer’s protocol (VECTOR, Burlingame, CA). Biotinylated dsDNA was confirmed by ELISA. Fifty microliters of aqua blue was used to exclude dead cells in about 25 million PBMCs on D14 post-vaccination. Next, 50-μL antibody cocktail containing anti-CD3-PerCP (OKT3), anti-CD14-APC-cy7 (HCD14), anti-CD19-BV421 (HIB19), anti-CD20-PE-cy7 (L27), anti-IgM-APC (G20-127), anti-IgG-PE (G18-145), and 15-μL biotinylated dsDNA (130 ng/μL) was surface stained for 1 h at 4 °C. To sort hemagglutinin (HA)-specific single B cells, 10 μL biotinylated HA (200 ng/μL, Emory University) was used. After three times washings with PBS, 50 μL of 1:1000 diluted streptavidin-FITC (BD Pharmingen) was added and incubated for 15 min at 4 °C. Antigen-specific IgG+ single B cells (CD3–CD14–CD19+CD20+IgG+ dsDNA+ or HA+) were identified and sorted into a 96-well PCR plate containing 10 μL of catching buffer per well using FACS Aria II cell sorter (BD Biosciences, San Jose, CA). The catching buffer contained 2 μL of 10× first strand buffer (Invitrogen, Carlsbad, CA), 2 μL of 0.1 M DTT (Invitrogen), 1 μL of RNase Out (Invitrogen), and 0.05 μl of igepal (Sigma-Aldrich, St. Louis, MO). The sorted cells were stored at − 80 °C for future uses, and flow cytometry data was analyzed by FlowJo software (Version 10.0.8).

### Single B cell immunoglobulin gene amplification and antibody expression

As described in previous studies [20], the frozen plates with sorted dsDNA-specific single B cells were thawed at room temperature, and reverse transcription was carried out by adding 3 μL of 50 μM random hexamers (Invitrogen), 1 μL of 10 mM dNTP mix (Invitrogen), and 1 μL of SuperScript III (Invitrogen) into each well. The mixture was incubated at 25 °C for 10 min, 50 °C for 50 min, and 85 °C for 5 min. IgH, Igκ, and Igλ genes were amplified from cDNA. All PCRs were performed in 96-well PCR plates in a total volume of 25 μL containing 5 μL of 5× HotStar HiFidelity PCR buffer, 1 μL of primer (20 μM), and 0.25 μL of HotStar HiFidelity Polymerase (Qiagen). Each round of PCR was initiated at 94 °C for 5 min, followed by 40 cycles of 94 °C for 30 s, 58 °C 60 s, and 72 °C for 1 min, followed by 72 °C for 10 min. The positive products were selected for direct sequencing or subsequently cloned into the corresponding expression vectors as previously described [21]. The recombinant plasmids were co-transfected into 293 T cells with equal amounts of paired heavy and light chains using Lipofectamine 3000 (Invitrogen). The full length of IgGs was expressed and purified using Protein A/G Agarose (ThermoFisher, Waltham, MA).

### ANA immunofluorescence

ANA immunofluorescence was analyzed following the manufacturer’s protocol (MBL, Des Plaines, IL). Briefly, Hep-2-coated slides were incubated with 25 μL of 1:40 diluted plasma for 30 min in a moist chamber at room temperature, then washed with PBS and incubated with 30-μL FITC-labeled goat anti-human IgG for 30 min. ANA-negative and ANA-positive controls were included in all experiments. After washing with PBS, samples were examined with a confocal microscope (Olympus BX61). The staining of patient samples was determined by comparison with the controls at equal exposure times.

### ELISA development for detection of antibodies against lipoteichoic acid of *Staphylococcus aureus*

Purified lipoteichoic acid (LTA) from *Staphylococcus aureus* (InvivoGen) was diluted at the concentration of 4 μg/mL in coating buffer (KPL, Milford, MA) and added to microtiter wells. The plate was incubated at 4 °C overnight. Microwells were washed three times with phosphate buffered saline wash buffer (PBS with 0.1% Tween 20) and blocked with PBS containing 3% bovine serum albumin (BSA) for 120 min at 37 °C. Plasma was diluted 1:50 for IgA anti-LTA antibody detection and 1:5000 for IgG anti-LTA antibody detection in PBS containing 3% BSA, and 100 μL of the dilution was added to each well. The plate was incubated at room temperature for 60 min with rotation at 450 rpm. Horseradish peroxidase (HRP)-labeled goat anti-human IgA or IgG was added to make a 1:2500 dilution in PBS containing 3% BSA. The plate was then incubated for 60 min at room temperature. After washing, 100 μL 2, 2′-azino-di (3-ethylbenzothiazoline-6-sulfonate) (ABTS) was added and incubated for 30 min, and finally, 405-nm emission was read within 30 min.

### Affinity purification of ANA from plasma

Antibodies were purified in plasmas of human subjects. Briefly, ANA IgG or IgM from the plasmas of HIV+ subjects was purified using NHS Mag Sepharose (GE Healthcare). Buffer of Hep-2 laryngeal carcinoma cell lysates (ATCC) was exchanged to PBS using ultra centrifugal filters (Amicon, EMD Millipore, Massachusetts), then covalently coupled to NHS magnetic beads. Plasma samples and binding buffer were mixed at a 1:1 ratio and incubated at 4 °C for 4 h in a column with ANA antigens immobilized on magnetic beads. The unbound fraction was removed, and the column was washed extensively with 50 mM Tris/150 mM NaCl. ANA was eluted sequentially with 0.1 M glycine/HCl buffer at pH 2.9 and concentrated using ultra centrifugal filters (Amicon, EMD Millipore, Massachusetts).

### Competitive ELISA

Hep-2 laryngeal carcinoma cell lysates (ATCC) were diluted in PBS and added to microtiter wells at 4 °C overnight. Microwells were then washed three times with PBS wash buffer (0.1% Tween 20). The reaction was blocked using blocking buffer (KPL, Milford, MA) for 120 min at 37 °C. Purified ANAs were diluted two times from 1:10. The antibody solution was incubated with influenza vaccine antigen at 1:1 ratio for 30 min at room temperature. Antibody solution in PBS at 1:1 ratio was used as a control. One hundred microliters of the reaction mixture was added to the wells coated with antigens and incubated at room temperature for 1 h. After washing for three times, goat anti-human IgM/IgG-conjugated HRP was added at a 1:5000 dilution in PBS containing 3% BSA and incubated for 60 min at room temperature. HRP-Streptavidin was added at a 1:1000 dilution in PBS containing 3% BSA, and then incubated for 30 min at room temperature. After washing, 100 μL ABTS was added and incubated for 30 min, and the plate was read at 405 nm emission within 30 min.

### Autoantigen microarray

Autoantigen microarrays were performed by Genomics and Microarray Core at the University of Texas Southwestern Medical Center. IgG autoantibodies from plasma were analyzed using 125-plex autoantigen arrays. Plasma samples were treated with DNase I, diluted to 1:50, and incubated with autoantigen arrays. Cy3-labeled anti-IgG was used to detect IgG autoantibodies, and the arrays were scanned with a GenePix® 4400A Microarray Scanner. The images were analyzed using GenePix 7.0 software. The averaged net fluorescent intensity of each autoantigen was normalized to internal controls, and scaling and centering (*x* − mean(*x*))/sd(*x*) were performed after log_10_ transformation.

### Plasma LPS level

Plasma LPS levels were measured by endpoint chromogenic limulus amebocyte lysate assays kit (Lonza, Basel, Switzerland) according to the manufacturer’s protocol. Samples were 1:10 diluted with endotoxin-free water and subsequently heated to 85 °C for 15 min to inactivate inhibitory proteins. Background was subtracted, and LPS levels were calculated based on standards.

### Gene expression analysis by microarray

In the current study, total B cells (purity > 97%) were isolated in PBMCs using B cell isolation kit (Miltenyi Biotec, Bergisch Gladbach, Germany). Total RNA from B cells was extracted using RNeasy Micro kit following the manufacturer’s protocol (Qiagen, Valencia, CA), quantified by Nanodrop 2000 (Nanodrop, Wilmington, DE), and qualified by Agilent 2100 Bioanalyzer (Agilent Technologies, Santa Clara, CA). RNA concentration more than 20 ng/μL was selected to perform Affymetrix GeneChip assays, including high ANA HIV+ subjects (*n* = 5) and low ANA HIV+ subjects (*n* = 9) on D0 and D7 post-vaccination. Affymetrix Human GeneChip U133 Plus 2.0 Array was used for RNA hybridization and labeling assay according to the manufacturer’s protocol (Affymetrix). The scanned images and probe signals were analyzed by GCOS (Affymetrix). All microarray data and statistical analyses were performed using R (version 3.3.1). Normalization was performed with RMA algorithm which included background adjustment and quantile normalization [22]. Selection of distinct gene expression was identified by *P* value less than 0.05. Biological profiles of distinct genes were analyzed and clustered using iPathway (Advaita, Plymouth, MI). All microarray data are available in the GEO database.

### Next-generation sequencing of the IGH repertoire

Next-generation sequencing (NGS) of the B cells receptor (BCR) repertoire was described in a previous study [19]. RT-PCR reactions were conducted using a set of nested sequence-specific primers covering the variable (V) and constant (C) regions. Illumina paired-end sequencing communal primer B was linked to each inside V region primer. Illumina paired-end sequencing communal primer A and a barcode sequence were linked to the reverse inside C-beta primer. cDNA was reverse transcribed from the total RNA samples using a mixture of forward V-beta and reverse C-beta primers and reagents from the RT-PCR kit (Qiagen). After the first round of amplification, the PCR products were purified using AMPure XP beads (Beckman Coulter, Indianapolis IN). The second-round PCR was performed using a set of communal primers that completed the Illumina adaptor sequences. Final products were purified and quantified by Nanodrop 2000, pooled in equimolar proportions, and followed by 300 bp paired-end sequencing on the Illumina MiSeq platform. Raw high-throughput sequencing reads were quality controlled, assembled, and filtered using pRESTO [23]. V(D)J germline segments were assigned using IMGT/HighV-QUEST (http://www.imgt.org/). Clonal clustering was performed using Change-O [24]. Clone abundance, diversity, and amino acid property were analyzed as described in previous studies [25]. Custom statistical analysis and plots were assessed and shown by R.

### Plasma circulating microbial 16S rDNA

Method of plasma 16S rDNA analysis was described in a previous study [26]. In the current study, bacterial DNA was extracted from 400 μL of plasma or endotoxin-free water controls using QIAamp UCP Pathogen Mini Kit according to the manufacturer’s instructions (Qiagen). To prevent batch-to-batch variation, all samples were sequenced at the same time. V4 variable region of bacterial 16S rDNA gene was amplified using PCR primers 515/806 in HotStarTaq Plus Master Mix (Qiagen) under the following conditions: 94 °C for 3 min, followed by 28 cycles of 94 °C for 30 s, 53 °C for 40 s, and 72 °C for 1 min, and a final elongation step at 72 °C for 5 min. Sequencing was performed at MR DNA on an Ion Torrent PGM following the manufacturer’s guidelines (Shallowater, TX, USA). Sequencing data was processed using a proprietary analysis pipeline (MR DNA). Briefly, sequences were depleted of barcodes and primers, short sequences less than 200 bp were removed, and sequences with ambiguous base calls and homopolymer runs exceeding 6 bp were also removed. Sequences were denoised and operational taxonomic units (OTUs) were defined clustering at 3% divergence (97% similarity) followed by removal of singleton sequences and chimeras. Final OTUs were taxonomically classified using BLASTn against a database derived from RDPII and NCBI (www.ncbi.nlm.nih.gov).

OTU tables and different levels of taxonomic tables were imported to R for statistical analysis. To remove potential bacterial 16S rDNA contamination from molecular biological reagents, we removed genera from experimental samples at the OTU-level if they were detected in the water controls. To measure the richness and evenness of a plasma microbiome community, Simpson index of diversity was calculated using Vegan package to measure α-diversity of each sample. ANCOVA model was used to assess the associations with Simpson diversity index, clinical and demographic characters, and levels of autoreactive antibody. The Bray-Curtis coefficient was calculated using the Vegan package to evaluate β-diversity and compositional dissimilarity among the microbial community. The relationship between β-diversity and autoreactive antibodies was assessed using permutational multivariate analysis of variance (PERMANOVA).

### Statistical analysis

Conventional measurements of central location and dispersion were used to describe the data, and differences in continuous measurements between groups were compared by non-parametric Mann-Whitney’s *U* tests. To explore associations between pairs of continuous variables, Spearman’s rank correlation was used. Comparison analysis was performed using R (version 3.3.1) or GraphPad Prism 6. All tests were two-sided, and *P* ≤ 0.05 was considered to denote statistical significance.

## Results

### Autoantibodies induced by seasonal influenza vaccination in HIV+ subjects

**Fig. 1:**
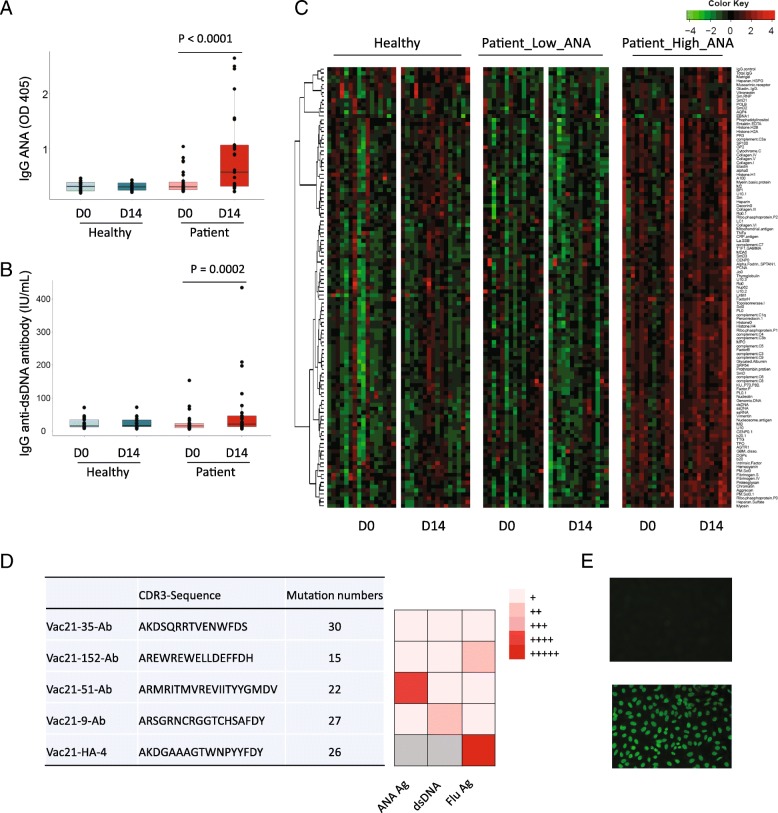
Autoantibodies generated after seasonal influenza vaccination in HIV-infected individuals. ELISA and autoantibody array were performed using plasma samples on D0 and D14 post-vaccination. Detection of plasma IgG ANA (**a**) and anti-dsDNA (**b**) autoantibodies from healthy (*n* = 16) and HIV+ (*n* = 26) subjects. **c** Detection of IgG autoantibodies by protein array containing 125 self-antigens from healthy (*n* = 16), HIV+ low ANA (*n* = 14), and HIV+ high ANA (*n* = 12) subjects. **d** Autoreactivity and polyreactivity of four anti-dsDNA mAbs (left margin, clone number, and CDR3 sequence) and one anti-influenza HA mAb (V21-HA-4) as a control by ELISA. (E) Polyreactivity of anti-dsDNA mAb and anti-influenza HA mAb against cellular nuclear antigens (Hep-2 cells) by immunofluorescent microscopy. Original magnification, × 40. Non-parametric Mann-Whitney *U* tests

Following immunization with the 2013–2014 seasonal influenza vaccine in 16 healthy controls and 26 ART aviremic HIV-infected subjects (Additional file 1: Table S1), we found that the IgG ANAs were increased in HIV+ subjects (*P* < 0.0001), but there was no change in healthy controls (*P* = 0.53) (Fig. 1a). To identify if other autoantibodies besides ANA were generated after immunization, we measured plasma IgG anti-dsDNA antibody, a pathologic autoantibody in systemic lupus erythematosus (SLE) [27]. Indeed, IgG anti-dsDNA antibodies showed the same pattern as IgG ANAs. After immunization, plasma IgG anti-dsDNA antibody levels were significantly increased in HIV-infected subjects (*P* = 0.0002), but not in healthy controls (*P* > 0.99) (Fig. 1b). Next, we examined the overall profile of autoantibodies using an autoantigen array containing 125 self-antigens that have previously been shown to induce autoantibodies [28]. HIV-infected subjects were stratified to high ANA (fold change of ANA on D14/D0 ≥ 2) (*n* = 12) and low ANA (fold change of ANA on D14/D0 < 2) (*n* = 14) groups. Consistent with the results from ANA and anti-dsDNA antibodies, the universal autoantibodies did not change in healthy controls on D14; however, the autoantibodies against a wide spectrum of autoantigens were significantly increased in response to influenza vaccination in HIV+ subjects on D14 (Fig. 1c).

### Autoantibodies generated from bystander B cell activation

To investigate the source of autoreactive antibodies induced by influenza vaccination, we purified ANA polyclonal antibodies from plasmas of HIV+ high ANA subjects and examined their binding abilities to nuclear antigens in the presence of influenza antigens by competitive ELISA. Little cross-reactivity was observed in IgG ANAs with influenza antigens (Additional file 1: Figure S1A). Similar to IgG ANA, the majority of IgM ANAs were not cross-reactive to influenza antigens (Additional file 1: Figure S1B). We also sorted dsDNA+IgG+ B cells from an HIV+ subject who had more than fourfold induction of IgG anti-dsDNA (D14/D0) (Additional file 1: Figure S1C). Two hundred twenty-six single dsDNA-specific IgG+ B cells were sorted. Heavy or light chains were successfully amplified from 147 single B cells. Eighty-nine recombinant monoclonal antibodies (mAbs) were generated from heavy chains with paired light chains, and 31 of those mAbs displayed substantial reactivity to dsDNA antigens. The average number of mutations per sequence among anti-dsDNA mAb was 16.4 (Additional file 1: Table S2). None of these mAbs was clonally related. Only four of them (12.9%) showed low-affinity cross-reactivity to influenza vaccine antigens (Fig. 1d), and the average number of mutations per sequence among these four mAbs was 23.5. We further measured the reactivity of these four anti-dsDNA mAbs with Hep-2 cell lysates, and with one mAb from influenza hemagglutinin (HA)-specific IgG+ B cells in the same donor set as a control, all of those four mAbs showed cross-reactivity (Fig. 1d, e, Additional file 1: Table S2). After the IgH and IgL chain genes of those polyreactive four mAbs were reverted back to their germline form, all four mAbs lost reactivity to dsDNA antigens and influenza vaccine antigens. These results suggest that those four out of 31 anti-dsDNA mAbs were from the non-reactive precursors, and the other vaccine-mediated autoantibodies were not generated via molecular mimicry of similar epitopes of influenza antigen or via polyreactivity of influenza-specific B cells. Thus, the autoantibodies should be generated independently with influenza antigen-specific antibodies, but from bystander B cells activation [11].

### Systemic microbial translocation was associated with ANA production in HIV+ subjects

**Fig. 2:**
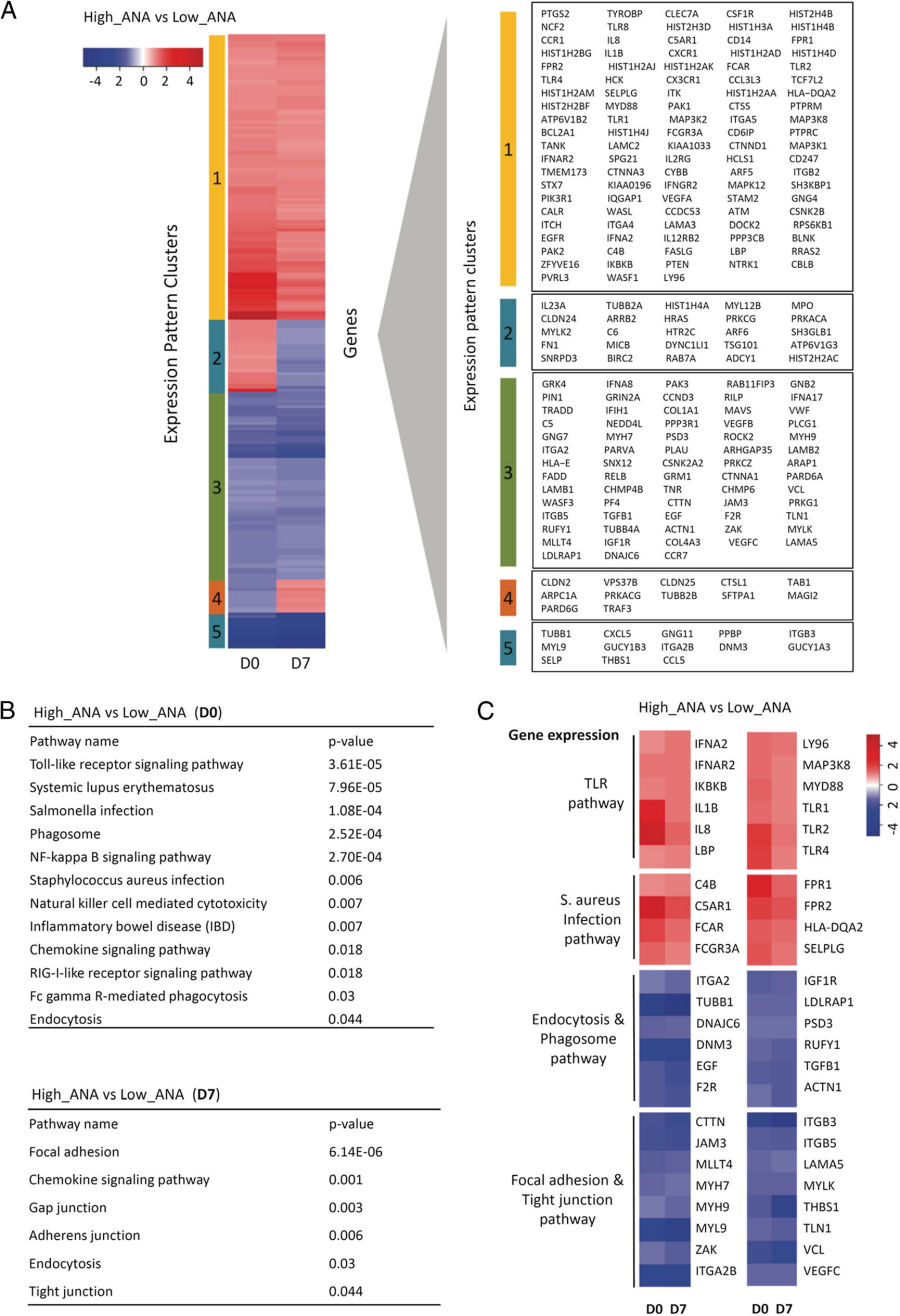
Gene expression profiles of purified B cells from HIV+ subjects with high ANA compared to low ANA production in response to influenza vaccination. The comparisons were analyzed by the ratios of gene expression in sorted total B cells from HIV+ high ANA (*n* = 5) vs low ANA (*n* = 9) subjects on D0 and D7 post-vaccination. **a** Heat map of fold changes in gene expression. Genes with similar patterns were grouped by clustering analysis (indicated by color bars on the left) and represented coherently changed genes in the clusters. **b** Representative immunological pathways enriched in coherently changed genes in HIV+ high ANA compared to low ANA subjects. **c** Expression of various gene encoding products in selected pathways showing increased (red) or decreased (blue) expression in HIV+ high ANA compared to low ANA subjects

To determine the mechanism of autoantibody generation in HIV+ subjects, RNA was isolated from purified total B cells and analyzed by microarray. Gene expression was shown after normalization (Additional file 1: Figure S2). Relative transcript abundance was shown as the ratio of gene expression from HIV+ high ANA group vs low ANA group (Fig. 2a). To better quantify this gene variation in a functional context, the biological pathways that associated with gene expression were characterized. A selected list of biological pathways with enriched transcripts was shown to reflect the significant differences between the two HIV+ groups (Fig. 2b).

The reduced gene expression of endocytosis and phagosome pathway in HIV+ high ANA subjects was compared to low ANA subjects, suggesting a decreased clearance of apoptotic debris along with immune complexes [29, 30]. Notably, HIV+ high ANA group showed decreased gene expression that related to focal adhesions, gap junction, adherens junction, and tight junction compared to those in low ANA group (Fig. 2b, c). The B cell transcriptome alterations may be the downstream of impaired mucosal barriers but need further investigation. In contrast, genes related to the TLR signaling pathway such as TLR1, TLR2, TLR4, and MyD88 were highly expressed in the HIV+ high ANA group compared to low ANA group (Fig. 2b, c). These TLR-associated genes play a role in sensing translocated microbial products and producing pro-inflammatory cytokines [31]. Similarly, IL-1β, IFN-α, and IL-8, which are downstream cytokines of TLR signaling pathway, were highly expressed in the HIV+ high ANA group compared to low ANA group (Fig. 2c). These results suggest that microbial translocation-related innate immune activation is associated with autoantibody production in response to influenza vaccination in HIV disease.

### Systemic microbial translocation and distinct B cell repertoire profiles in autoantibody induction in HIV+ subjects

**Fig. 3:**
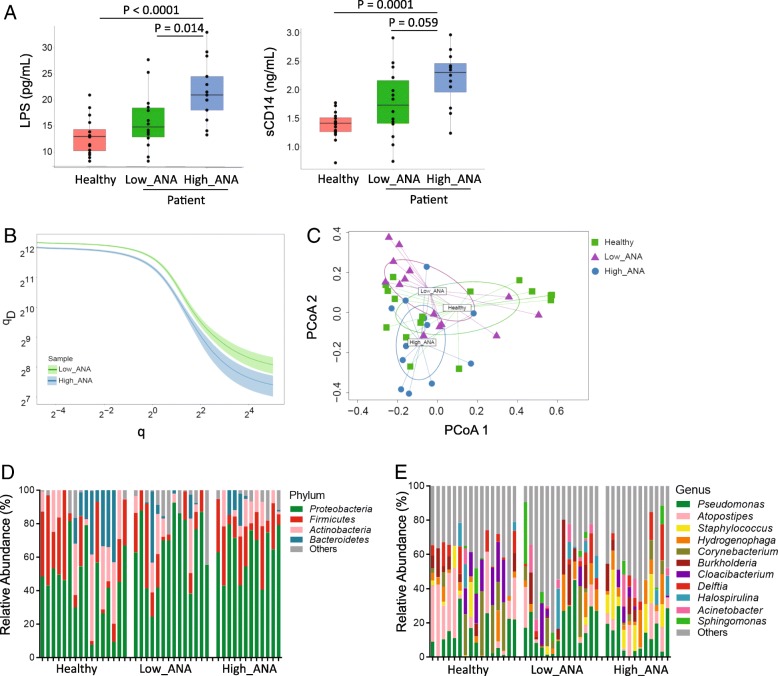
Systemic microbial translocation and distinct B cell repertoire profiles in autoantibody induction in HIV+ subjects. **a** Plasma LPS and sCD14 level were analyzed at baseline (D0) from the three study groups. **b** Sorted B cells on D14 post-vaccination were analyzed by BCR repertoire, the clonal diversity of B cells from HIV+ groups shown by the Hill diversity index (qD, *y* axis). The median diversity score over all resampling realizations and 95% percentile were plotted as a line and a shaded background. All samples were randomly sampled to 4000 sequences for each resampling realization to correct for variations in sequencing depth. **c** PCoA plot of Bray-Curtis dissimilarity comparing baseline plasma bacterial 16s microbiome communities. **d** The average relative abundance of each bacteria at the phylum level. **e** The average relative abundance of each bacteria at the genus level

The results of B cell gene expression profiles by microarray indicate that microbial translocation may play a critical role in the generation of autoantibodies in ART-treated HIV disease. We therefore examined plasma LPS level, a marker of systemic microbial translocation, among the three study groups. Elevated plasma LPS level was observed in HIV+ high ANA group compared to the other two groups (Fig. 3a). Importantly, plasma LPS level at baseline was positively correlated with both IgG ANA (*r* = 0.49, *P* = 0.014) and IgG anti-dsDNA (*r* = 0.44, *P* = 0.029) levels after immunization (Additional file 1: Figure S3A, D14/D0). To establish evidence for direct chronic LPS stimulation, we measured plasma soluble CD14 (sCD14), which secreted by CD14+ monocyte/macrophages and can bind LPS, and plasma LPS binding protein (LBP), which is produced by gastrointestinal and hepatic epithelial cells in response to LPS stimulation. The results showed higher levels of plasma sCD14 in HIV+ high ANA group compared to the other two groups (Fig. 3a), but no difference of plasma LBP was observed (Additional file 1: Figure S3B).

TLR activation has been shown to promote clonal expansion of autoreactive cells via TLR/B cell receptor (BCR) signal transduction [32, 33]. BCR/TLR dual signals in mature B cells can enhance B cell activation and directly initiate humoral autoimmunity [34]. To test whether BCR repertoires are different between ANA low and ANA high HIV+ subjects, we performed high-throughput sequencing of heavy chain variable (V), joining (J), and diversity (D) gene segments (VDJ) using purified B cell RNA in three donors from each HIV+ group on D14 after immunization. Sequencing was obtained and analyzed in each subject. The overall diversity of total B cells had a lower general sequence diversity index (qD) [35] in HIV+ high ANA group compared to low ANA group (Fig. 3b). The contraction in diversity was shown in both the total number of clones (q →0) and clonal dominance (q →∞) (Fig. 3b). We then investigated BCR clonal diversity in each isotype separately, and the same pattern was observed (Additional file 1: Figure S3C). These results showed that B cells from HIV+ high ANA group have significantly more focused number of clones and indicated promoted clonal expansion compared to repertoires of HIV+ low ANA group. Highly diverse CDR3 region is thought to be the key determinant for antigen specificity and antibody recognition, and we found the median charges of CDR3 region in total IgA and IgG were increased in HIV+ high ANA group compared to low ANA group, whereas the median charges of CDR3 region in IgM were similar between the two HIV+ groups (Additional file 1: Figure S3D). These results were consistent with the previous study that autoreactive antibodies are more likely to have positively charged CDR3 region [20] and indicated that the B cells selection checkpoint in the GC from HIV+ high ANA group is abnormal [1, 36].

### Plasma microbiome in autoantibody induction in HIV+ subjects

Studies on microbiome have shown that microbial species play a role in regulating host immune system [37]. In the current study, we are interested in systemic plasma microbiome and which microbial species affects autoantibody production in HIV. We detected and analyzed bacterial 16S rDNA in plasma. To prevent the batch-to-batch variation, the samples from high ANA, low ANA, and healthy controls were sequencing at the same time. The OTUs detected in the water controls (Additional file 1: Table S3) were removed from results in the plasma samples. We analyzed Gini Simpson diversity index to compare diversity of overall microbial community and did not observe a significant difference between any two study groups (α-diversity, Additional file 1: Figure S3E). To compare compositional dissimilarity among the microbial communities (β-diversity), the Bray-Curtis algorithm was applied to calculate distances between samples and shown in principal coordinate analysis (PCoA) plots. Again, we did not observe a significant difference in β-diversity between the two HIV+ groups (PERMANOVA, *P* > 0.05) (Fig. 3c). We further analyzed plasma microbiome on different taxonomic levels. At the phylum-level, plasma microbiome was dominated by *Proteobacteria* (mean ± SD, 44.9% ± 22.0% vs 67.3% ± 19.7% vs 56.3% ± 21.0%), *Firmicutes* (22.0% ± 17.8% vs 14.7% ± 18.3% vs 21.7% ± 13.8%), and *Actinobacteria* (18.4% ± 19.1% vs 6.0% ± 9.2% vs 12.4% ± 10.0%) in healthy controls, HIV low ANA, and high ANA subjects, respectively (Fig. 3d). At the genus level, a significant increase in *Staphylococcus* was found in HIV+ high ANA group compared to low ANA group (Fig. 3e).

### Plasma translocation of *Staphylococcus* correlates with autoantibody production

**Fig. 4:**
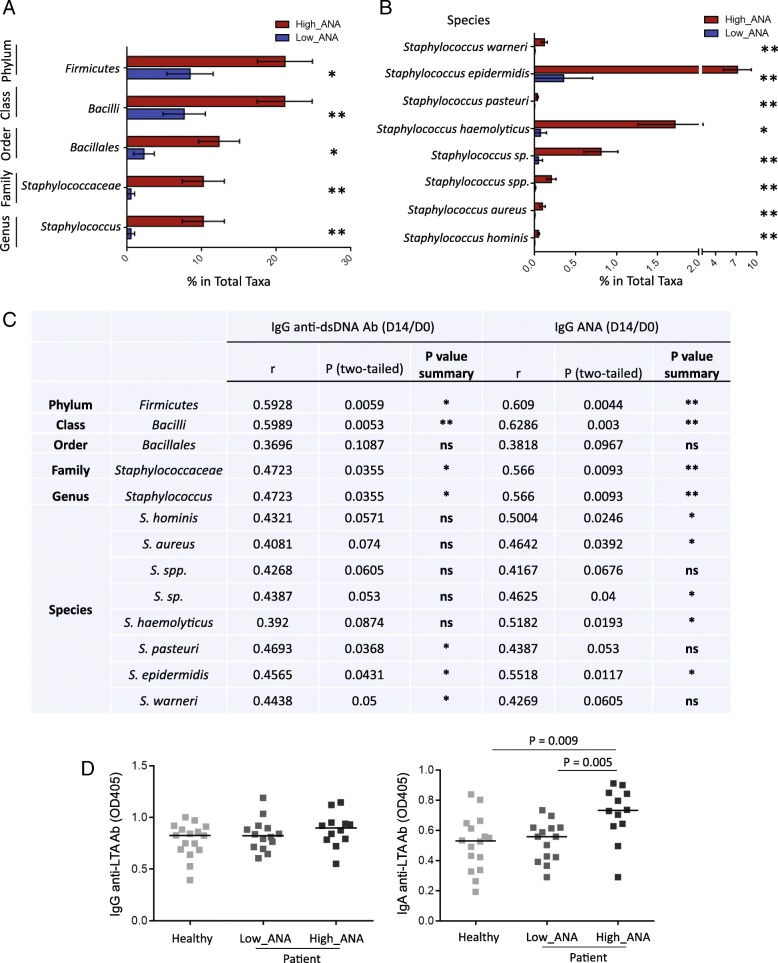
Plasma translocation of *Staphylococcus* correlates with autoantibody production. Significant increases in relative abundance of *Staphylococcus*-associated taxa from the phylum, class, order, family, genus levels (**a**) to the species level (**b**) in high ANA compared to low ANA HIV+ groups. Bar graphs represent mean percent taxa abundance ± SD. **c** Correlations between autoantibody induction and frequencies of *Staphylococcus*. **d** Baseline plasma levels of anti-*Staphylococcus aureus* LTA IgG and IgA. Mann-Whitney *U* (unpaired) and Spearman’s rank tests. *P < 0.05; **P < 0.01

Given these results, we wondered if increased plasma level of *Staphylococcus* taxonomic group was related to autoantibody induction in HIV+ subjects. We compared *Staphylococcus* taxa between the two HIV+ groups and observed that HIV+ high ANA group had increased *Staphylococcus*-associated taxa from the phylum to species level (Fig. 4a, b). Additionally, there were significant positive correlations between frequencies of plasma *Staphylococci* species and fold-changes of both ANA and anti-dsDNA IgG production in HIV+ subjects (Fig. 4c). These results indicate that *Staphylococci* may play a critical role in mediating autoantibody production in response to vaccination in ART-treated HIV+ subjects. Lipoteichoic acid (LTA, a TLR2 ligand) is the major constituent of cell walls from gram-positive bacteria. To verify the enrichment of *Staphylococci* in HIV+ high ANA group, we evaluated plasma level of antibody against LTA purified from *Staphylococcus aureus* and found plasma anti-LTA IgA but not IgG level was increased in HIV+ high ANA group compared to the other two groups (Fig. 4d). These results suggest that *Staphylococcus* infection or stimulation occurring mainly through the mucosal route [38] may play a role in systemic humoral immune perturbations in HIV disease.

### *Staphylococcus* induces autoantibody production, GC responses, and autoimmunity in mice

**Fig. 5:**
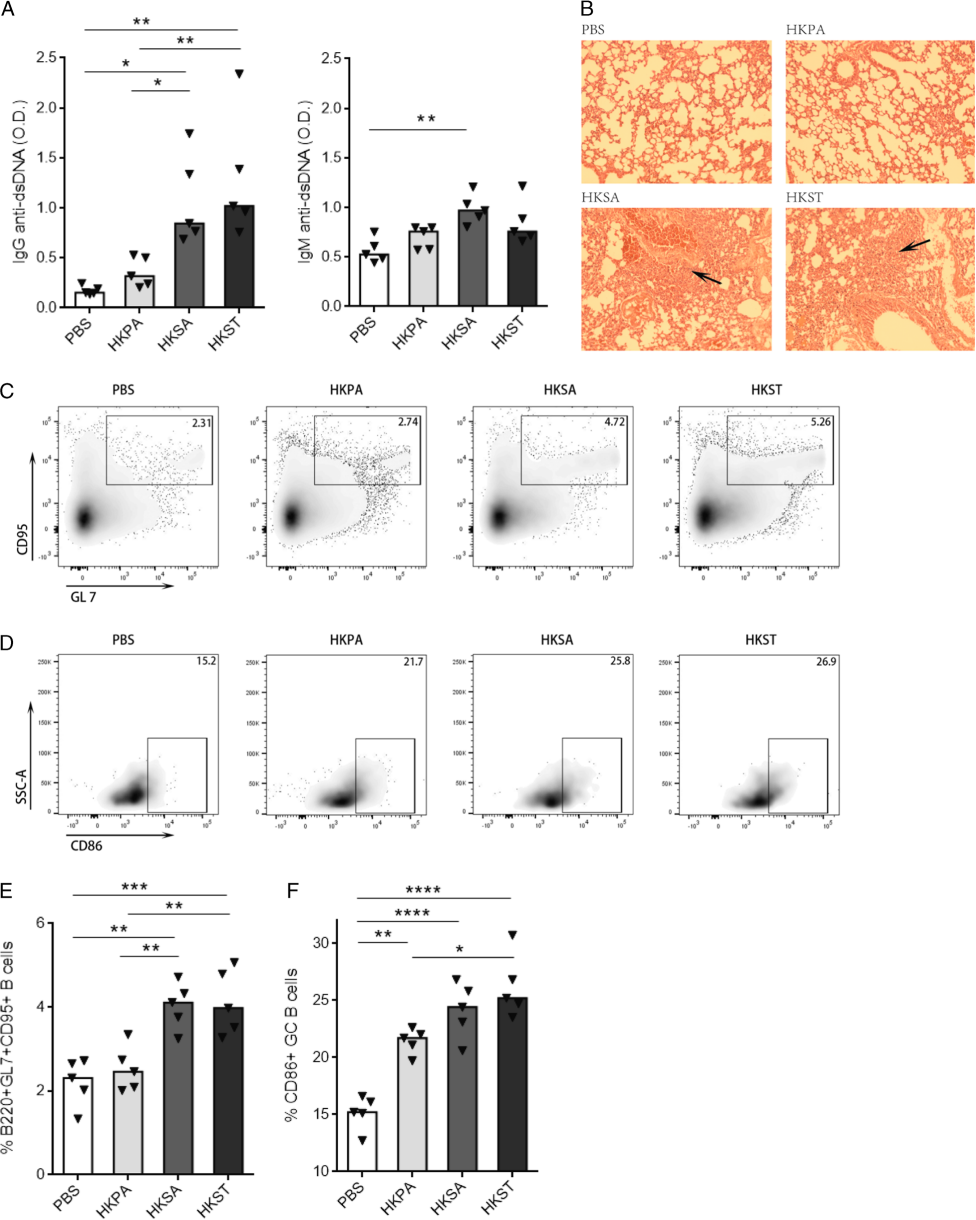
*Staphylococcus* induced autoantibody and autoimmunity. C57BL/6 mice were injected with PBS, or HKPA, or HKSA, or HKST twice a week for 4 weeks and following one time a week for 8 weeks by intraperitoneal (i.p.) route, *n* = 5 per group. Figures were shown as scatter plot with median. **a** Anti-dsDNA autoantibodies were measured by ELISA in sera, and optical density (O.D.) indicates ELISA color change and the presence of anti-dsDNA autoantibodies. **b** The alveolar septa widened and inflammatory cell infiltration induced by HKSA or HKST were shown by H&E-staining. **c** The percentages of germinal center cells (CD95+GL7+) from peripheral lymph nodes are shown in gated CD19+B220+ B cells. **d** The percentages of CD86+ cells are shown in gated germinal center B cells. **e** Proportions of GC B cells increased in the lymph nodes of HKSA and HKST groups compared to PBS or HKPA group. (F) The frequency of CD86+ GC B cells increased in the lymph nodes of mice after receiving HKST, HKSA, or HKPA. One-way ANOVA test, *p < 0.05, **p < 0.01, ***p < 0.001, ****p < 0.0001

To further evaluate whether *Staphylococcus* is responsible for the elevated autoimmune response found in some HIV+ individuals, we tested the ability of *Staphylococcus* to induce autoimmunity in mice. *Salmonella* has been proved to induce autoantibodies [39] and was set as a positive control. Although *Pseudomonas* is the one of predominant taxa in HIV+ individuals, the high prescience of *Pseudomonas had no correlation with* autoantibody production. Therefore, *Pseudomonas* was chosen as the negative control for testing the ability of microbiome to induce autoantibody production. To test this, 6-week-old healthy C57BL/6 mice were injected with phosphate buffered saline (PBS), heat-killed *Salmonella typhimurium* (HKST), heat-killed *Pseudomonas aeruginosa* (HKPA), or heat-killed *Staphylococcus aureus* (HKSA) twice a week for 4 weeks and then once a week for 8 weeks by intraperitoneal (i.p.) route. As expected, mice injected with PBS remained negative for autoantibodies. However, mice injected with HKST or HKSA produced robust anti-dsDNA autoantibodies, whereas mice injected with HKPA produced low levels of autoantibodies (Fig. 5a). Besides the autoantibody production, we observed the alveolar septa widened and inflammatory cell infiltration after injected with HKST or HKSA (Fig. 5b), and the disintegration of intestinal villi in response to HKST (Additional file 1: Figure S4A).

To investigate the effects of HKSA on adaptive immune cells in vivo, we measured B cell response in peripheral lymph nodes. Similar to HKST, HKSA induced significantly GC B cell differentiation and CD86 expression on GC B cells when compared with HKPA and PBS control (Fig. 5c–f), suggesting that HKSA and HKST promote GC responses in mice. Th17 cells are a pathogenic effector CD4 T cell lineage that plays critical roles in the pathogenesis of some autoimmune diseases [40]. In view of the high level of anti-dsDNA antibodies in the response to HKST and HKSA, it was logical to ask whether Th17 would also show difference after being treated with HKSA or HKSA. Although the frequency of IL-17A-producing T cells in the spleen showed no difference after being treated with different bacteria (Additional file 1: Figure S4B), increased percentage of IL-22+ in the spleen memory CD4+ T cells was observed in mice being treated with HKSA when compared with those treated with HKPA (Additional file 1: Figure S4C). Intriguingly, compared with PBS control group, the CD25+CD69+ memory CD4+ T cells and the IFN-γ+ CD4+ T cells were significantly increased in mice treated with HKPA but not in those treated with HKSA and HKST (Additional file 1: Figure S4D-4E). Those results indicated that *Staphylococcus* is capable to induce autoimmunity in mice through enhancing GC response and autoantibody production.

## Discussion

Although both thymus-dependent (TD) and thymus-independent (TI) B cell responses are decreased in HIV-infected individuals prior to ART [41–43], most B cell defects in HIV infection are reversed after administration of ART [44]. An intriguing insight from this work was that the influenza antigen elicited autoantibodies in aviremic ART-treated HIV+ subjects. We further investigated the mechanism and cellular origins accounting for this observation.

Some autoantibodies can recognize both self-antigens and microbial epitopes after infection [45]. However, results from the competitive antigen binding experiment and mAbs showed that very limited number of autoantibodies displayed cross-reactivity. It indicated that the cellular origins of autoantibodies derived from the bystander B cells [46]. We further characterized gene expression patterns in B cells from HIV+ high ANA subjects compared to low ANA subjects by microarray and identified marked differences in genes related to microbial translocation, such as the TLR pathway, *Staphylococcus aureus* infection pathway, endocytosis and phagosome pathway, and focal adhesion and tight junction pathway (Fig. 2b, c). Tight junctions have been shown to play a role in regulating lung mucosal epithelial barrier function [47, 48], which relate to mucosal permeability and alveolar fluid clearance. Subsequently, we found that both quantity and quality of plasma translocated bacterial products at baseline were associated with autoantibody production in response to vaccination in HIV+ subjects.

Microbiota plays a role in regulating host immune system development, homeostasis, and immunity against infection [49–51]. The microbiota composition in the system and mucosal surface in human is extremely variable and is related to host disease states [52, 53]. The dominant species on the skin, in the oral cavity, or in the gastrointestinal tract differs significantly [54]. Systemic microbial translocation may be the key drivers for microbial product-mediated inflammation, innate immune activation, and immune perturbation [55, 56], but their roles in human health remain largely unknown. Plasma level of LPS, a marker of bacterial load [12], was increased in the high ANA HIV+ individuals compared to the other two study groups. The bacterial load in the plasma in the high ANA HIV+ individuals was far lower than the level in the plasma from patients with sepsis. Moreover, healthy individuals had lower LPS and sCD14 compared with HIV individuals. The plasma 16S rDNA analysis showed that although there was no difference in the diversity of microbiota composition within each of the healthy controls and HIV+ subjects, there was a significant increase in *Staphylococcus*-associated taxa from the phylum to the species level in HIV+ high ANA subjects compared to low ANA subjects. Notably, even after long-term ART treatment, skin or tissue infection of *Staphylococcus aureus* is still common in HIV-infected patients [57]. *Staphylococcus aureus* mainly colonizes on the skin and mucosa in human [58]; therefore, it is possible that enriched plasma *Staphylococcus aureus* found in some HIV+ subjects is derived from skin contamination during venipuncture, even the skin was wiped by alcohol prior to a blood draw. However, increased plasma level of IgA against LTA from *Staphylococcus aureus* was observed in HIV+ subjects with high ANA group compared to controls (Fig. 4d), which cannot be explained by skin contamination during venipuncture. Moreover, there was a direct correlation between the enrichment of *Staphylococcus*-associated taxa and vaccine-induced autoantibody production. Additional experiments on C57BL/6 mice found that heat-killed *Staphylococcus aureus* directly induced autoantibodies.

TLRs play critical roles in host immunities by recognizing and responding to microbial pathogens; they also mediate inflammatory responses and are involved in autoreactive antibody induction in autoimmune diseases [16–18, 59]. Microarray analysis of B cells has revealed that high ANA HIV+ subjects had upregulation of *IL1B* and *IL23* genes, which favor immune activation and are essential for disease development in several models of autoimmune disease [60]. TLRs/MyD88 signaling in B cells has been proposed to increase B cell migration to the GC dark zones [61] and contribute to their sustainment in the GC [62]. Meanwhile, TLRs may cooperate in conjunction with the BCR to play a function in the process of B cell negative selection. It has been suggested that alterations in BCR and TLR signaling pathways result in a defective central checkpoint and a failure to counter-selection developing autoreactive B cells in the bone marrow [63]. Moreover, B cell activation enhanced by BCR/TLR dual signals can directly initiate humoral autoimmunity [34]. In our study, we found more focused and promoted clonal expansion of B cells and increased charges of CDR3 region in IgA and IgG from HIV+ high ANA group compared to repertoires of HIV+ low ANA group. Moreover, treatment with inactivated *Staphylococcus aureus* and *Salmonella typhimurium* in C57BL/6 mice resulted in increased autoantibodies, B cells in the GC, and GC B cell activation. Our mice study showed that heat-inactivated *Staphylococcus aureus* promoted germinal center (GC) responses. *Staphylococcus* protein A (SpA) is a *Staphylococcus* surface protein [64]. SpA drives polyclonal B cell expansion, induces cell death, and enhances the short-lived extrafollicular response [64], and *Staphylococcus* lipoteichoic acid (LTA), on the surface of *Staphylococcus*, binds to TLR2 and activates TLR2 cell signaling pathway. TLRs can promote clonal expansion of autoreactive B cells after receiving BCR signals following antigen stimulation, which may provide the initial step in the generation autoantibodies [65].

In summary, our study has revealed that *Staphylococcus* can promote B cell activation and drive autoantibody production in ART-treated HIV+ subjects under immunological recovery. It may uncover a potential mechanism linking microbial translocation and autoimmunity.

## Additional file

Additional file 1: **Table S1**. Clinical characteristics and baseline immune parameters. **Table S2**. Repertoire and reactivity of 31 mAbs from dsDNA+IgG+ B cells in one HIV+ subject displayed substantial reactivity to dsDNA antigens. **Table S3**. The OUT in water control. **Figure S1**. Cross-reactivity of purified IgG and IgM ANA to influenza vaccine antigens by competitive ELISA. **Figure S2**. Variation in gene expression. **Figure S3**. Systemic microbial translocation, B cell repertoire profiles, and plasma microbiome in autoantibody induction in HIV+ subjects. **Figure S4**. H&E-stained sections of small intestine and T cells response from spleen of C57BL/6 mice after treated with PBS, HKPA, HKSA, or HKST. (PDF 3448 kb)

## Abbreviations

ANA: Anti-nuclear antibody
ART: Antiretroviral therapy
BCR: B cells receptor
GC: Germinal center
HA: Influenza hemagglutinin
LPS: Lipopolysaccharides
mAbs: Monoclonal antibodies
TLR: Toll-like receptor
