# Supplementary material for: Systemic translocation of Staphylococcus drives autoantibody production in HIV disease
Source: Microbiome. 2019 Feb 14;7:25. doi: 10.1186/s40168-019-0646-1 (PMC6376754; doi:10.1186/s40168-019-0646-1)
Supplement: Supplementary file 1 — Table S1. Clinical characteristics and baseline immune parameters. Table S2. Repertoire and reactivity of 31 mAbs from dsDNA+IgG+ B cells in one HIV+ subject displayed substantial reactivity to dsDNA antigens. Table S3. The OUT in water control. Figure S1. Cross-reactivity of purified IgG and IgM ANA to influenza vaccine antigens by competitive ELISA. Figure S2. Variation in gene expression. Figure S3. Systemic microbial translocation, B cell repertoire profiles, and plasma microbiome in autoantibody induction in HIV+ subjects. Figure S4. H&E-stained sections of small intestine and T cells response from spleen of C57BL/6 mice after treated with PBS, HKPA, HKSA, or HKST. (PDF 3448 kb) [file 40168_2019_646_MOESM1_ESM.pdf]

Supplementary Tables

	HIV-<br>(n = 16)	HIV+/low ANA<br>(n = 14)	HIV+/high ANA<br>(n = 12)	P value (two HIV+ groups)
Age	38 (33-55)	43 (26-52)	43 (36-54)	0.41
Sex (male/female)	5/11 (31.3%)	10/4 (71.4%)	7/5 (58.3%)	0.68
CD4 counts	710 (429-936)	572 (476-800)	566 (442-798)	0.91
Nadir CD4 counts		348 (211-469)	258 (189-469)	0.70
Duration of ART		5 (4-6)	5 (3-6)	0.79
%annexin V+ CD4	19 (13-40)	30 (19-33)	32 (21.5-43)	0.69
%CD38+ mCD4	14.3 (10.1-16.8)	20.1 (16.1-27.8)	11.8 (4.6-20.7)	0.05
%ki67+ CD4	1.1 (0.7-1.8)	2.4 (1.7-4.2)	2.5 (1.9-3.2)	0.97
B cell counts	176 (74-221)	146 (110-185)	245 (113-321)	0.04
%annexin V+ B	11.6 (6.5-16.6)	14 (12.7-21.4)	30.1 (19.5-39.8)	0.003
%ki67+ B	0.9 (0.8-1.5)	1.6 (0.9-2.8)	1.2 (0.9-1.8)	0.38

Table S1. Clinical characteristics and baseline immune parameters

Data are medians (interquartile ranges)

P value: comparisons of P value between the two HIV+ groups on D0

							mAb reactive with Ag		
Sequence ID	V-GENE	J-GENE	D-GENE	CDR3-Sequence	Mutation numbers	%Mutation rate	dsDNA	flu vaccine	Nuclear Ag
VH-134-Ab	IGHV4-30-4*01 F	IGHJ4*02 F	IGHD3-22*01 F	AEQPYYYDTSALN	21	7.14	+	−	NA
VH-178-Ab	IGHV3-30*03 F, or IGHV3-30*18	IGHJ4*02 F	IGHD4-23*01 ORF	AGGNDYGDHSPLDY	7	2.41	+ +	−	NA
VH-156-Ab	IGHV3-30*03 F, or IGHV3-30*18	IGHJ1*01 F	IGHD6-13*01 F	AKDLGIAAAVAQD	12	4.05	+	−	NA
VH-169-Ab	IGHV3-23*01 F, or IGHV3-23D*0	IGHJ4*02 F	IGHD3-22*01 F	AKDLLGSGYYYAFDY	6	2.03	+ +	−	NA
VH-35-Ab	IGHV3-11*01 F	IGHJ5*01 F, or IGHJ5*02 F	IGHD4-23*01 ORF	AKDSQRRTVENWFDS	30	10.14	+	+	+
VH-20-Ab	IGHV3-23*04 F	IGHJ4*02 F	IGHD6-13*01 F	AKVGHSPFFDY	10	3.39	+	−	NA
VH-27-Ab	IGHV1-18*01 F	IGHJ5*02 F	IGHD3-10*01 F	ARAQWPGDWLDP	23	7.80	+	−	NA
VH-5-Ab	IGHV4-38-2*02 F	IGHJ3*01 F, or IGHJ3*02 F	IGHD2-8*01 F	ARDFYDIVLRLYAPEEAFDL	19	6.42	+	−	NA
VH-25-Ab	IGHV3-30-3*01 F	IGHJ3*01 F	IGHD3-10*01 F	ARDQEERSPLVRGIFNDAFDV	30	10.14	+ + +	−	NA
VH-2-Ab	IGHV1-69*01 F, or IGHV1-69D*0	IGHJ5*02 F	IGHD6-13*01 F	ARDSGSWFKFDP	27	9.12	+	−	NA
VH-59-Ab	IGHV1-3*01 F	IGHJ3*02 F	IGHD2-15*01 F	ARDVDPGATPRAFDI	4	1.35	+	−	NA
VH-48-Ab	IGHV3-21*01 F	IGHJ4*02 F	IGHD4-17*01 F	AREGRRLRGDHDEDFDF	23	7.77	+ +	−	NA
VH-152-Ab	IGHV4-39*01 F	IGHJ4*02 F	IGHD1-26*01 F	AREWREWELLDEFFDH	15	5.05	+	+ +	+
VH-30-Ab	IGHV1-69*01 F, or IGHV1-69*11	IGHJ1*01 F	IGHD1-1*01 F	ARFDQMDRLTTRDFQG	15	5.10	+	−	NA
VH-218-Ab	IGHV1-8*01 F	IGHJ4*02 F	IGHD3-3*01 F	ARGRVDDFSSGCSGF	17	5.74	+	−	NA
VH-79-Ab	IGHV1-46*01 F, or IGHV1-46*03	IGHJ3*02 F	IGHD1-1*01 F	ARHQGRDAFDI	10	3.40	+ +	−	NA
VH-51-Ab	IGHV6-1*01 F	IGHJ6*02 F	IGHD3-10*01 F	ARMRITMVREVIITYYGMDV	22	7.26	+	+	+ + + +
VH-24-Ab	IGHV3-30*04 F, or IGHV3-30-3*	IGHJ5*02 F	IGHD6-6*01 F	ARPSTIAAGNWFDP	10	3.40	+	−	NA
VH-36-Ab	IGHV1-69*12 F	IGHJ4*02 F	IGHD6-19*01 F	ARQAVTGSLDY	14	4.76	+	−	NA
VH-9-Ab	IGHV4-61*03 F	IGHJ4*02 F	IGHD2-15*01 F	ARSGRNCRGGTCHSAFDY	27	9.28	+ +	+	+
VH-49-Ab	IGHV1-46*01 F, or IGHV1-46*03	IGHJ6*02 F	IGHD2-15*01 F	ARSIVVVVAVTPDYYYGIDV	10	3.40	+	−	NA
VH-91-Ab	IGHV4-30-4*01 F	IGHJ4*02 F	IGHD3-22*01 F	ARVGNSRLSAHEPFDY	20	6.71	+ +	−	NA
VH-74-Ab	IGHV1-18*04 F	IGHJ2*01 F	IGHD3-16*01 F	ARVRTGDQRRGNYAMVPYFDL	25	8.47	+	−	NA
VH-18-Ab	IGHV1-69*01 F, or IGHV1-69D*0	IGHJ6*02 F	IGHD5-12*01 F	ARVVDWLANQDYYYQGMDV	16	5.42	+	−	NA
VH-64-Ab	IGHV4-4*02 F	IGHJ4*02 F	IGHD3-9*01 F	ARVWYYDILTGYQRGYYFDY	7	2.37	+	−	NA
VH-55-Ab	IGHV3-30-3*01 F	IGHJ4*02 F	IGHD6-13*01 F	ASCWVSYTSNWHGDYLDY	11	3.75	+	−	NA
VH-26-Ab	IGHV1-18*04 F	IGHJ4*02 F	IGHD3-22*01 F	ASPTLWESSGFYAQYFFDD	18	6.21	+	−	NA
VH-77-Ab	IGHV4-39*01 F	IGHJ4*02 F	IGHD6-13*01 F	ASVPIPGYSSSPAASFDY	5	1.69	+ +	−	NA
VH-7-Ab	IGHV4-39*01 F	IGHJ1*01 F	IGHD4-23*01 ORF	CYGGNPWD	11	3.79	+	−	NA
VH-28-Ab	IGHV4-39*01 F	IGHJ4*02 F	IGHD3-22*01 F	TGGHKALYYDSSGFYWGRIFNY	16	5.50	+ +	−	NA
VH-164-Ab	IGHV3-11*04 F	IGHJ3*01 F, or IGHJ3*02 F	IGHD3-22*01 F	VRIISSSYLYDGFNL	27	9.18	+	−	NA

−: OD405 < 0.15
+: OD405 > 0.3
++: OD405 > 0.6
+++: OD405 > 0.9
++++: OD405 > 1.2
+++++: OD405 > 2.5
NA：didn’t detect

Table S2. Repertoire and reactivity of 31 mAbs from dsDNA+IgG+ B cells in one HIV+ subject displayed substantial reactivity to dsDNA antigens.

	Plasma sample	Water control
**Average Observed OUTs**	93	30
**Average Copy Numbers**	30647	16908

						Average Copies		Average percentage in total OTU (%)	
**Phylum**	**Class**	**Order**	**Family**	**Genus**	**Species**	**Sample**	**Water control**	**Sample**	**Water control**
proteobacteria	alphaproteobacteria	sphingomonadales	sphingomonadaceae	sphingomonas	sphingomonas mali	66	593	0.216	3.504
					sphingomonas spp.	159	4	0.519	0.024
	betaproteobacteria	burkholderiales	burkholderiaceae	ralstonia	ralstonia insidiosa	15	48	0.048	0.284
			comamonadaceae	delftia	delftia acidovorans	46	36	0.152	0.210
					delftia lacustris	20	26	0.066	0.154
					delftia sp.	570	908	1.859	5.367
					delftia spp.	74	52	0.240	0.305
					delftia tsuruhatensis	254	165	0.829	0.976
				alicycliphilus	alicycliphilus spp.	113	470	0.369	2.777
				comamonas	comamonas testosteroni	20	53	0.064	0.313
				diaphorobacter	diaphorobacter sp.	4	21	0.012	0.121
				hydrogenophaga	hydrogenophaga spp.	1453	3648	4.742	21.573
				hylemonella	hylemonella spp.	47	172	0.154	1.017
				pelomonas	pelomonas aquatica	50	148	0.162	0.872
	gammaproteobacteria	pseudomonadales	pseudomonadaceae	pseudomonas	pseudomonas amygdali	77	194	0.252	1.147
					pseudomonas fluorescens	11	57	0.036	0.334
					pseudomonas gessardii	30	135	0.097	0.795
					pseudomonas libanensis	9	27	0.028	0.157
					pseudomonas lini	8	11	0.026	0.062
					pseudomonas migulae	84	132	0.275	0.778
					pseudomonas poae	273	629	0.891	3.717
					pseudomonas putida	797	1240	2.599	7.331
					pseudomonas rhodesiae	16	45	0.052	0.263
					pseudomonas sp.	158	496	0.516	2.931
					pseudomonas spp.	707	807	2.306	4.770
					pseudomonas straminea	1	14	0.004	0.083
					pseudomonas tolaasii	42	3	0.137	0.018
					pseudomonas veronii	624	1929	2.038	11.406
		xanthomonadales	xanthomonadaceae	stenotrophomonas	stenotrophomonas spp.	310	377	1.010	2.230
deinococcus_thermus	deinococci	thermales	thermaceae	thermus	thermus spp.	67	214	0.219	1.263
					thermus thiopara	361	1262	1.179	7.461
firmicutes	bacilli	lactobacillales	carnobacteriaceae	atopostipes	atopostipes sp.	1285	3003	4.192	17.758

Table S3. The OUT in water control

Supplementary Figures

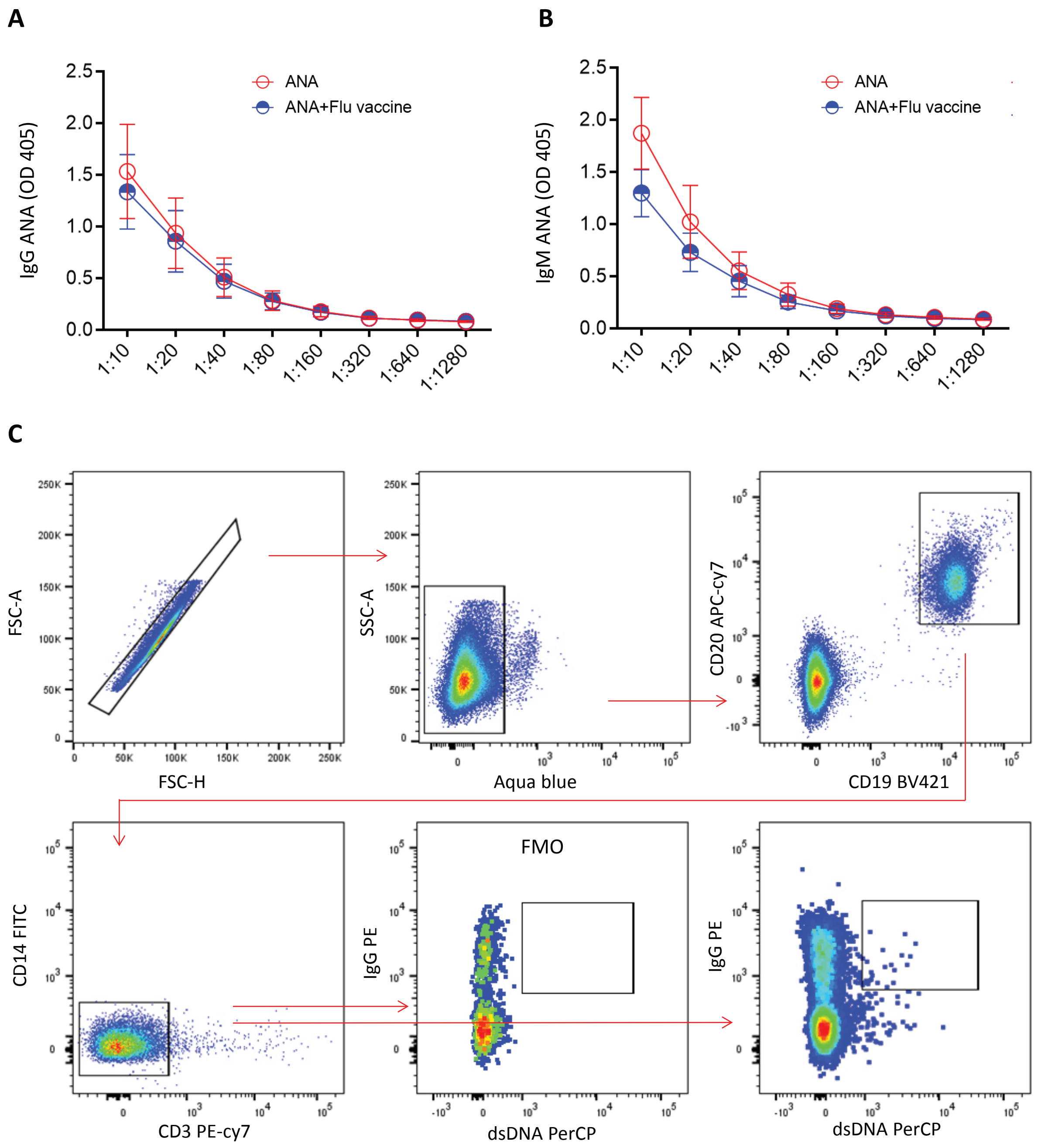

Figure S1. Cross reactivity of purified IgG (A) and IgM ANA (B) to influenza vaccine antigens by competitive ELISA. Polyclonal ANA was purified from plasmas of four high ANA HIV+ subjects on D14 post-vaccination. Purified ANA was tested its binding ability to nuclear antigens in the

presence or absence of influenza vaccine antigens. (C) Gating strategy for sorting single dsDNA-specific IgG+ B cells.

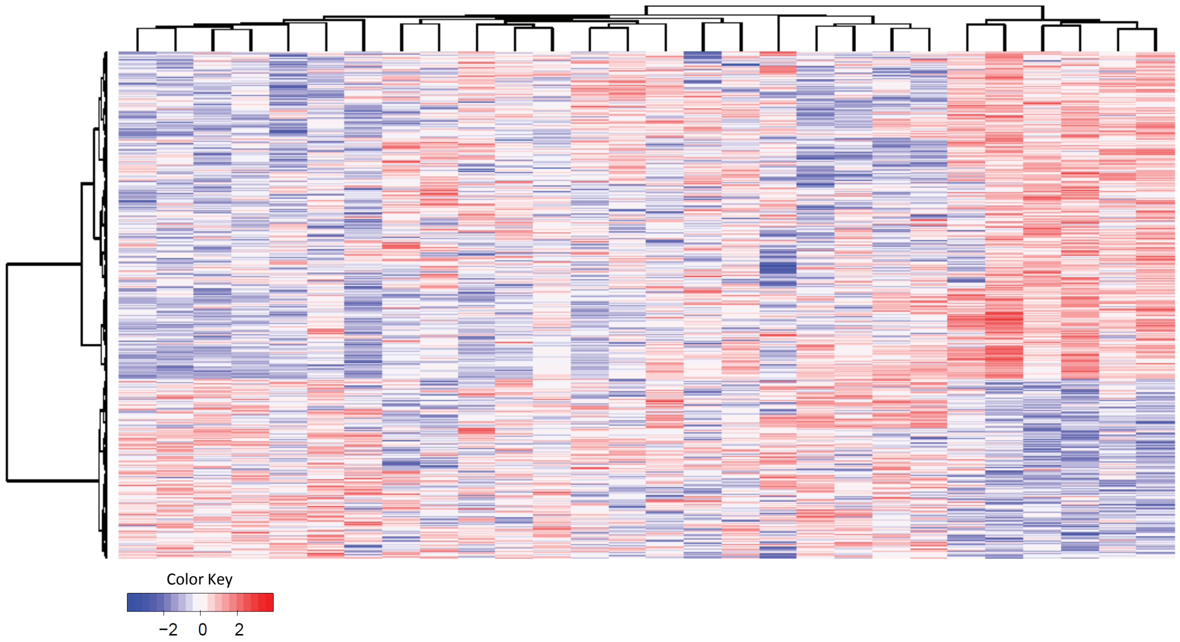

Figure S2. Variation in gene expression. Hierarchical-clustering of the 1000 most variable genes.

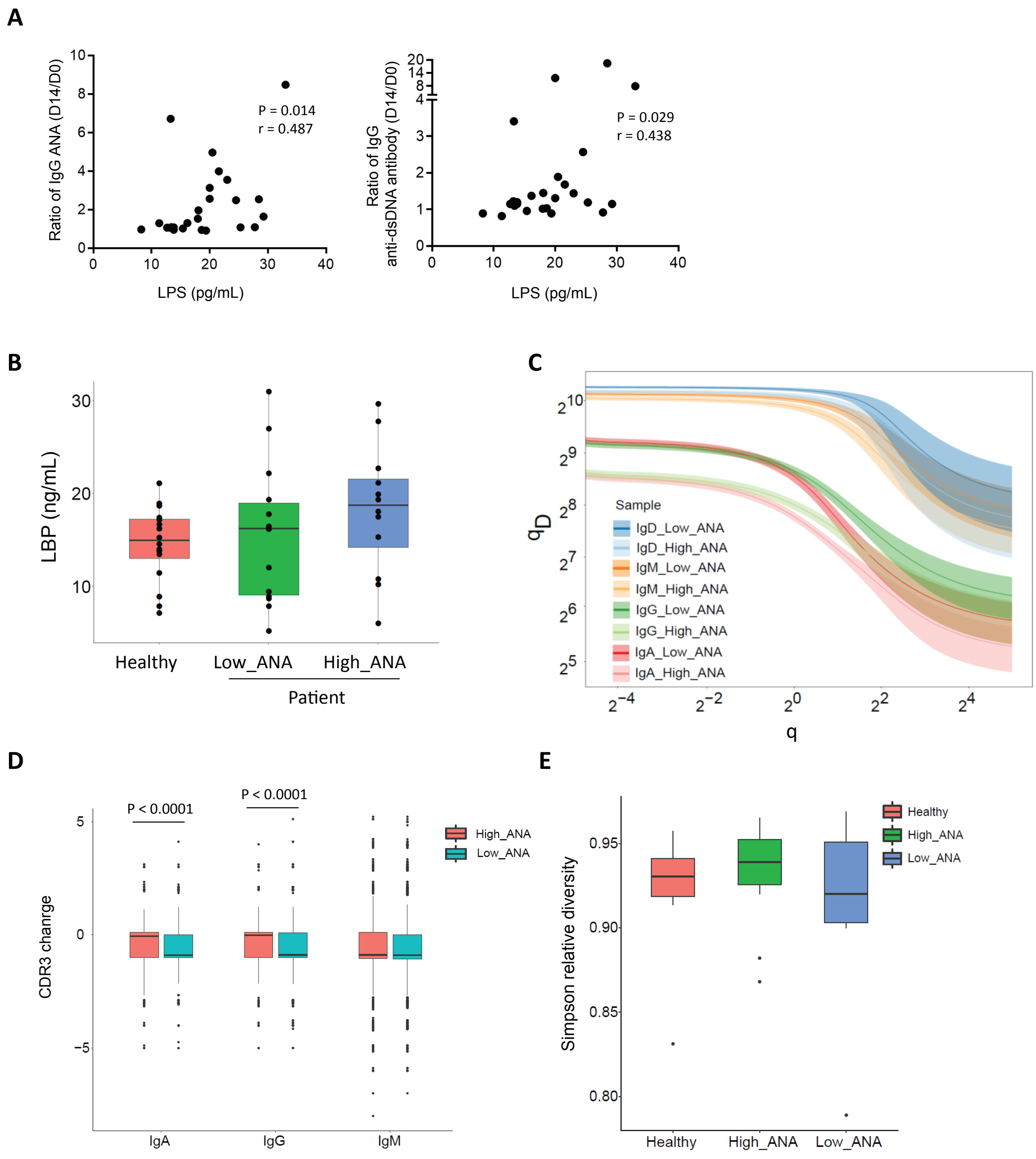

Figure S3. Systemic microbial translocation, B cell repertoire profiles, and plasma microbiome in autoantibody induction in HIV+ subjects. (A) Direct correlations between baseline plasma LPS level and IgG autoantibody induction by vaccination (D14/D0). (B) Plasma LBP level were

analyzed at baseline (D0) from the three study groups. (C) The clonal diversities of IgA, IgG, IgM, and IgD-positive B cells shown by the Hill diversity index (qD, y axis) within each HIV+ group. The median diversity score over all resampling realizations and 95% percentile were plotted as a line and a shaded background. All samples were randomly downsampled to 1000 sequences for each resampling realization to correct for variations in sequencing depth. (D) The median CDR3 charges of IgA, IgG, and IgM in the two HIV+ groups. (E) Gini Simpson diversity index (α-diversity) to compare diversity of overall microbial community from the three study groups.

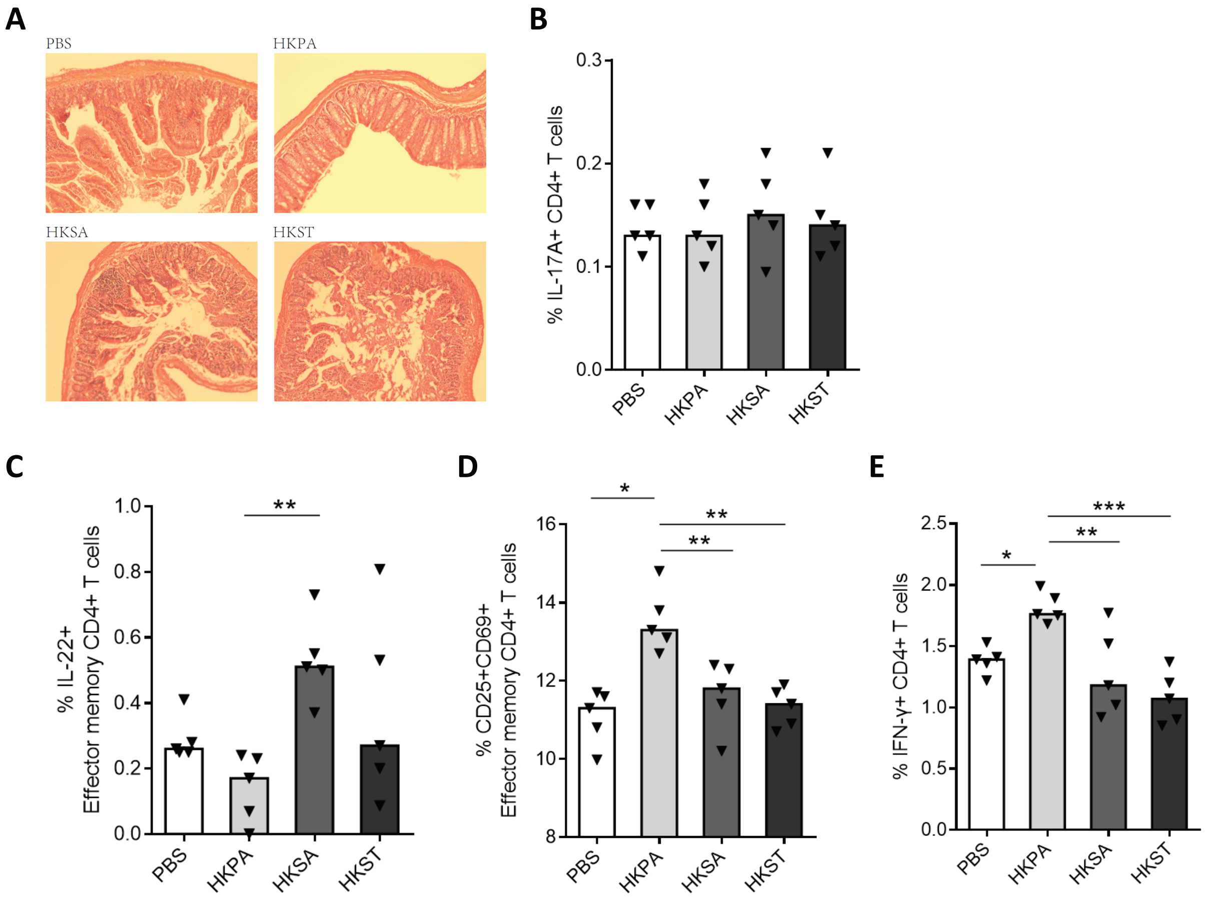

Figure S4. H&E-stained sections of small intestine and T cells response from spleen of C57BL/6 mice after treated with PBS, HKPA, HKSA, or HKST. (A) Disintegration of intestinal villi in HKST treated C57BL/6 mice. H&E-stained sections of small intestine after treatment. (B) Proportions of Th17 cells in the spleen showed no change when compared within four groups. (C) The frequency of IL-22+ memory ($CD44^{hi}CD62^{lo}$) CD4+ T cells increased in the spleen of HKSA group compared to HKPA group. (D) The frequency of CD25+CD69+ memory CD4+ T cells increased in the spleen of HKPA group compared to other groups. (E) The frequency of IFN-γ CD4+ T cells in the spleen of mice in all groups, IFN-γ staining were performed after 4

hours *ex vivo* stimulation with PMA and ionomycin in the presence of Brefeldin A. One-way ANOVA test, $*p < 0.05$, $**p < 0.01$, $***p < 0.001$, $****p < 0.0001$.
